# Association Between Time-Weighted Haemoglobin A1c and Longitudinal Changes in Growth Parameters Among Children With Type 1 Diabetes: A Retrospective Cohort Study

**DOI:** 10.7759/cureus.117044

**Published:** 2026-09-28

**Authors:** Fadi Ayyash, Sondos Harahshih, Hadeel Alqurieny, Asmaa M Al Quraan, Waed Itewi, Aseel Nimri, Osama Al Fanek, Saif Batarseh, Abdelrazaq A Al-Yasin

**Affiliations:** 1 Paediatric Endocrinology, Jordanian Royal Medical Services, Amman, JOR; 2 Paediatrics, Jordanian Royal Medical Services, Amman, JOR; 3 Endocrinology, Queen Rania Children’s Hospital, Amman, JOR; 4 Paediatric Surgery, Jordanian Royal Medical Services, Amman, JOR; 5 Endocrinology and Diabetes, King Hussein Medical City, Amman, JOR; 6 Paediatric Endocrinology, King Hussein Medical City, Amman, JOR

**Keywords:** child growth and development, glycated haemoglobin (hba1c), insulin regimen, suboptimal glycaemic control, type 1 diabetes mellitus (t1d)

## Abstract

Background: Managing early-onset type 1 diabetes requires balancing glycaemic control with developmental milestones. The relationship between cumulative glycaemic exposure and auxological outcomes in children has not been well characterised using time-weighted haemoglobin A1c (HbA1c).

Objective: This study explored the longitudinal association between time-weighted average HbA1c and changes in height-for-age, weight-for-age, and body mass index z-scores among children diagnosed with type 1 diabetes.

Methods: A retrospective cohort study included children aged 2-14 years with type 1 diabetes and ≥18 months of follow-up (n=200). The primary exposure was time-weighted average HbA1c. Outcomes were changes in height-for-age z-score (HAZ), weight-for-age z-score (WAZ), and BMI z-score (BMI-z). Linear mixed-effects models adjusted for age, sex, diabetes duration, insulin regimen, and pubertal status.

Results: Mean age was 9.5 ± 2.8 years, with 105 (52.5%) female subjects. Mean diabetes duration was 4.8 ± 3.2 years. A significant negative association was found between time-weighted average HbA1c and ΔHAZ (β = -0.15, 95% CI: -0.26 to -0.04, p < 0.01). Positive associations were identified with ΔWAZ (β = 0.08, 95% CI: 0.01-0.15, p<0.05) and ΔBMI-z (β = 0.10, 95% CI: 0.03-0.17, p<0.01). At baseline, suboptimal glycaemic control (HbA1c > 7.5%) was present in 116 (58.0%) of participants. The negative effect on HAZ was most pronounced in children with a diabetes duration >5 years (β = -0.22, p = 0.003) and in those with a baseline HbA1c >9% (β = -0.28, p < 0.001).

Conclusions: Lower time-weighted HbA1c, reflecting early and sustained glycaemic control, is associated with enhanced linear growth and a more favourable weight profile in children with type 1 diabetes.

## Introduction

Type 1 diabetes is one of the most common chronic endocrine disorders in childhood, with diagnosis often occurring during critical periods of growth [1, 2]. Haemoglobin A1c (HbA1c) reflects glycaemic control and predicts microvascular complications [3]. However, in children, treatment goals extend beyond metabolic parameters to ensure normal physical development [4].

Insulin acts as an anabolic hormone collaborating with the growth hormone-IGF-1 axis; chronic hyperglycaemia disrupts this pathway and can impair linear growth [5, 6]. Conversely, intensive insulin therapy has been associated with excessive weight gain [7]. The relationship between cumulative glycaemic exposure and auxological outcomes has not been well characterised using time-weighted HbA1c [8].

This study therefore aimed to evaluate the association between time-weighted average HbA1c, as a measure of cumulative glycaemic exposure, and longitudinal changes in height-for-age z-score (HAZ), weight-for-age z-score (WAZ), and body mass index z-score (BMI-z) over an 18-month observation period in children with type 1 diabetes. The term "early" in this context refers to glycaemic control during the 18-month observation period, which began at a relatively early stage of diabetes (mean duration 4.8 years) and during a critical period of childhood growth.

## Materials and methods

Ethics statement

This study received ethical approval from the Institutional Review Board (IRB) of the Jordanian Royal Medical Services (Registration Number: 5_16/2025) on November 24, 2024. On February 23, 2026, the Jordanian Royal Medical Services Educational and Training Directorate provided final authorisation for the dissemination and publication of data. The study was conducted in accordance with the ethical principles outlined in the Declaration of Helsinki. The IRB granted a waiver for individual informed consent due to the retrospective, anonymised nature of the research.

Study design and setting

A retrospective cohort study was conducted at the Paediatric Endocrinology Unit, Queen Rania Abdullah Hospital for Children, Royal Medical Services, Amman, Jordan, covering the period from January 2023 to May 2025. Longitudinal studies are particularly suited for assessing changes in auxological parameters over time, as they allow for the analysis of within-patient trajectories and the identification of predictors of growth outcomes [9]. The study is reported in accordance with the STROBE (Strengthening the Reporting of Observational Studies in Epidemiology) guidelines for observational studies [10].

Study population

The study screened the medical records of all children with type 1 diabetes who attended the paediatric endocrinology unit during the study period for eligibility. Patients were eligible for inclusion if they were aged 2 to 14 years at enrolment, had a confirmed diagnosis of type 1 diabetes, had a minimum follow-up of 18 months, and had at least three HbA1c and three anthropometric measurements during follow-up. Patients were excluded if they had conditions affecting growth (such as coeliac disease, hypothyroidism, or genetic syndromes), other chronic illnesses affecting growth or metabolism, or incomplete medical records. Following the application of these criteria, the final analytical cohort comprised 200 patients. The participant flow diagram is presented in Figure 1.

**Figure 1:**
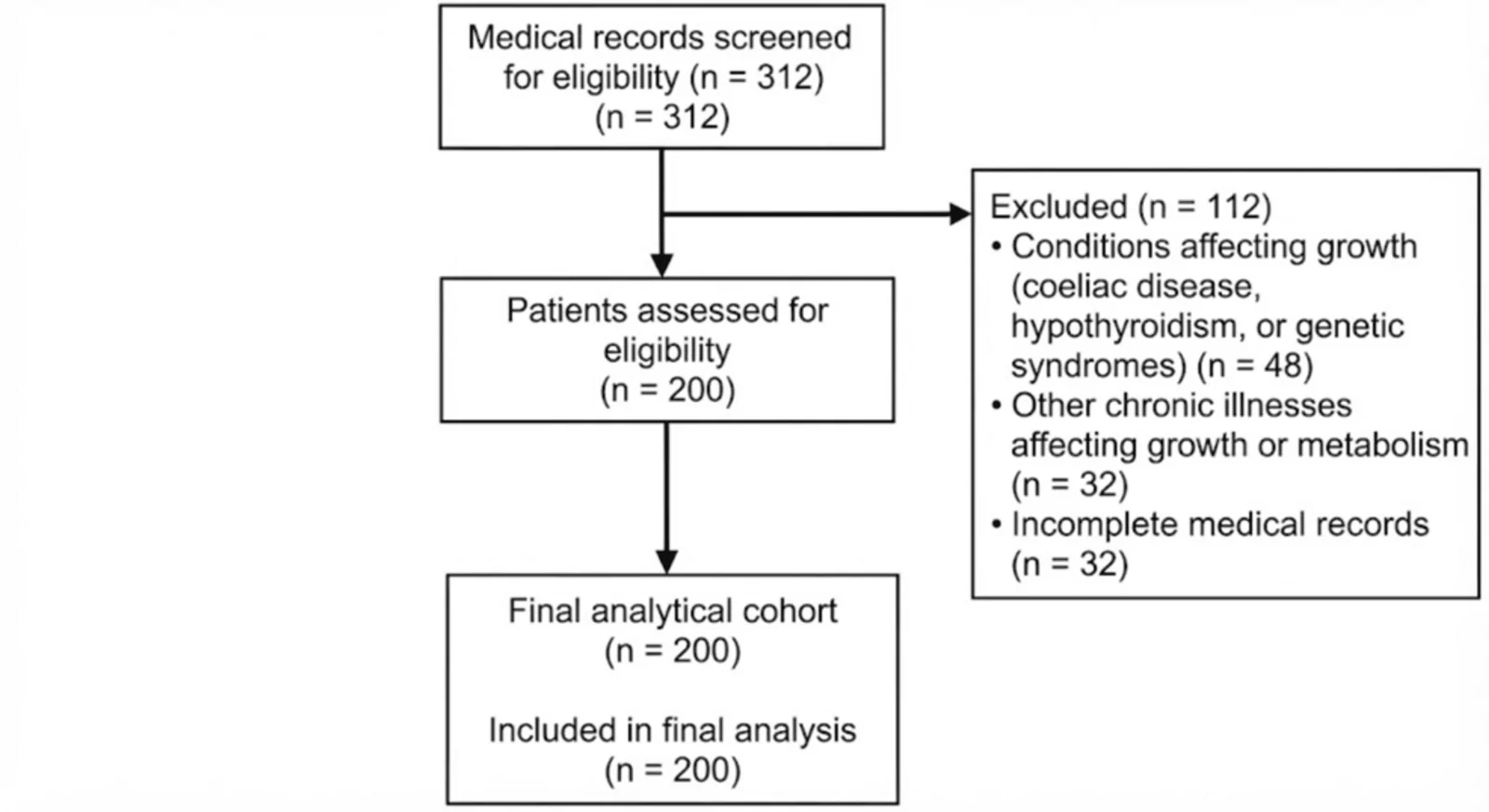
Participant flow diagram. This figure illustrates the participant screening, eligibility assessment, exclusion, and final cohort allocation process. A total of 312 medical records were screened, of which 112 were excluded: 48 due to conditions affecting growth (coeliac disease, hypothyroidism, or genetic syndromes), 32 due to other chronic illnesses affecting growth or metabolism, and 32 due to incomplete medical records. The remaining 200 patients met all inclusion criteria and constituted the final analytical cohort.

Data collection

Data were extracted from electronic medical records using a standardised data collection form. Variables collected included demographic and clinical data (age, sex, age at diagnosis, diabetes duration, pubertal status, and insulin regimen). Pubertal status was assessed using the Tanner staging system (Tanner stages I-V) based on a documented clinical examination by the treating paediatric endocrinologist at each clinic visit. For statistical adjustment, pubertal status was categorised as prepubertal (Tanner stage I) versus pubertal (Tanner stages II-V). When Tanner staging was not explicitly documented, pubertal status was inferred from age and documented growth parameters using standard paediatric criteria. A sensitivity analysis excluding patients with inferred pubertal status was performed to confirm the robustness of our findings. Glycaemic data included all HbA1c values recorded during the 18-month observation period. The time-weighted average HbA1c (TWA-HbA1c) was calculated using the trapezoidal rule:

$\mathrm{TWA\mathrm{-}HbA1c} = \frac{ \displaystyle \sum_i \left[ \frac{\mathrm{HbA1c}_i + \mathrm{HbA1c}_{i+1}}{2} \times (t_{i+1}-t_i) \right] }{ \displaystyle \sum_i (t_{i+1}-t_i) }$

where $\mathrm{HbA1c}_i$ represents the HbA1c value at time i, and $\mathrm{t}_i$ represents the time of measurement.

This approach accounts for unequal intervals between HbA1c measurements. The time-weighted average HbA1c was calculated exclusively from measurements obtained during the defined 18-month follow-up period and does not include measurements from the time of diagnosis or any period prior to the observation window. "Baseline HbA1c" refers to the first available HbA1c measurement at the beginning of the 18-month observation period, not the HbA1c at the time of type 1 diabetes diagnosis. Anthropometric data included height and weight measurements, which were converted to WHO z-scores: HAZ, WAZ, and BMI-z. Data were extracted using a systematic approach, consistent with model-driven data collection methodologies described in the literature [11, 12].

Outcome measures

The main results were the change in height-for-age z-score (ΔHAZ), the change in weight-for-age z-score (ΔWAZ), and the change in BMI z-score (ΔBMI-z) from the start of the study to 18 months.

Statistical analysis

Data were analysed using IBM SPSS Statistics for Windows, version 25 (IBM Corp., Armonk, NY, USA), with a two-tailed p-value of less than 0.05 considered statistically significant. Descriptive statistics were presented as mean ± standard deviation for continuous variables and as frequencies with percentages (n, %) for categorical variables. Linear mixed-effects models were employed to examine the association between time-weighted average HbA1c and changes in auxological outcomes. These models analysed all repeated anthropometric measurements obtained during the 18-month observation period (at baseline, 6 months, 12 months, and 18 months), rather than only the baseline-to-18-month change. The mixed-effects models accounted for within-patient correlation across repeated measures, handled unbalanced data due to missed visits, and allowed for the estimation of longitudinal trajectories of HAZ, WAZ, and BMI-z over time. The beta coefficients represent the average change in each z-score per 1% increase in time-weighted average HbA1c across all repeated measurements. Models were adjusted for age, sex, diabetes duration, insulin regimen, and pubertal status. The sample size of 200 patients was deemed adequate based on the rule of thumb requiring at least 10 events per variable for reliable regression analysis. Subgroup analyses were stratified by diabetes duration (>5 years vs. ≤5 years) and baseline HbA1c (>9% vs. ≤9%). While the current study focused on longitudinal associations, future research may benefit from decision curve analysis to evaluate the clinical utility of HbA1c-based prediction models for growth outcomes.

## Results

A total of 200 children were included in the final analysis. Baseline characteristics are summarised in Table 1. The mean age was 9.5 ± 2.8 years, with 105 (52.5%) female subjects. The mean diabetes duration was 4.8 ± 3.2 years. Baseline HbA1c was 8.1 ± 1.5%, with 116 (58.0%) having suboptimal control (>7.5%). Premixed insulin was used in 112 (56.0%), multiple daily injections in 87 (43.5%), and a hybrid regimen involving both premixed insulin and multiple daily injections at different time points in 1 patient (0.5%).

**Table 1:** Baseline demographic and clinical characteristics (N=200). SD, standard deviation; HbA1c, glycated haemoglobin; MDI, multiple daily injections.

Variable	Value
Age (years), Mean ± SD	9.5 ± 2.8
Female, n (%)	105 (52.5)
Diabetes duration (years), Mean ± SD	4.8 ± 3.2
Baseline HbA1c (%), Mean ± SD	8.1 ± 1.5
HbA1c >7.5%, n (%)	116 (58.0)
Premixed insulin, n (%)	112 (56.0)
Multiple daily injections (MDI), n (%)	87 (43.5)

Z-score changes over 18-month follow-up

Table 2 presents the baseline, follow-up, and mean change data for all three z-scores over the 18-month observation period. The height-for-age z-score (HAZ) declined from -0.42 ± 0.95 at baseline to -0.58 ± 1.02 at follow-up, representing a mean reduction of -0.16 ± 0.32 (p < 0.001), indicating a significant attenuation of linear growth relative to age-matched norms over the study period. In contrast, weight-for-age z-score (WAZ) increased from 0.15 ± 0.88 at baseline to 0.28 ± 0.92 at follow-up, with a mean increase of +0.13 ± 0.28 (p < 0.001), reflecting a trend toward disproportionate weight gain. Similarly, BMI z-score (BMI-z) rose from 0.32 ± 0.91 at baseline to 0.48 ± 0.95 at follow-up, with a mean increase of +0.16 ± 0.30 (p<0.001), demonstrating a tendency toward excess adiposity accrual. These divergent trajectories-declining linear growth with concurrent weight gain-indicate relative changes in linear growth and adiposity but do not represent clinical stunting or obesity as defined by established diagnostic criteria.

**Table 2:** Z-score changes over 18 months follow-up (N=200). SD, standard deviation; HAZ, height-for-age z-score; WAZ, weight-for-age z-score; BMI-z, body mass index z-score.

Z-Score	Baseline (Mean ± SD)	Follow-up (Mean ± SD)	Mean change (Δ)	p-value
Height-for-age (HAZ)	-0.42 ± 0.95	-0.58 ± 1.02	-0.16 ± 0.32	<0.001
Weight-for-age (WAZ)	0.15 ± 0.88	0.28 ± 0.92	+0.13 ± 0.28	<0.001
BMI z-score (BMI-z)	0.32 ± 0.91	0.48 ± 0.95	+0.16 ± 0.30	<0.001

When divided by glycaemic control, children with optimal control (HbA1c ≤7.5%) showed a smaller drop in HAZ (ΔHAZ: -0.09 ± 0.25 vs. -0.21 ± 0.35, p=0.004) and a smaller rise in BMI-z (ΔBMI-z: +0.09 ± 0.22 vs. +0.20 ± 0.34, p=0.006) than those with suboptimal control (HbA1c >7.5%). These findings underscore the protective effect of early and sustained glycaemic control on maintaining appropriate growth trajectories.

Longitudinal associations

Linear mixed-effects models (Table 3) revealed significant associations between time-weighted average HbA1c and auxological changes. These models analysed all repeated anthropometric measurements obtained during the 18-month observation period (at baseline, 6 months, 12 months, and 18 months), rather than only the baseline-to-18-month change. 

**Table 3:** Linear mixed-effects model – association between time-weighted HbA1c and auxological changes. Models adjusted for age, sex, diabetes duration, insulin regimen, and pubertal status. CI, confidence interval; HAZ, height-for-age z-score; WAZ, weight-for-age z-score; BMI-z, body mass index z-score.

Outcome	β coefficient (95% CI)	p-value
Δ Height-for-age z-score (HAZ)	-0.15 (-0.26 to -0.04)	<0.01
Δ Weight-for-age z-score (WAZ)	0.08 (0.01-0.15)	<0.05
Δ BMI z-score (BMI-z)	0.10 (0.03-0.17)	<0.01

A significant negative association was found between time-weighted average HbA1c and ΔHAZ (β = -0.15, 95% CI: -0.26 to -0.04, p < 0.01), indicating that higher cumulative glycaemic exposure was associated with reduced linear growth (Figure 2).

**Figure 2:**
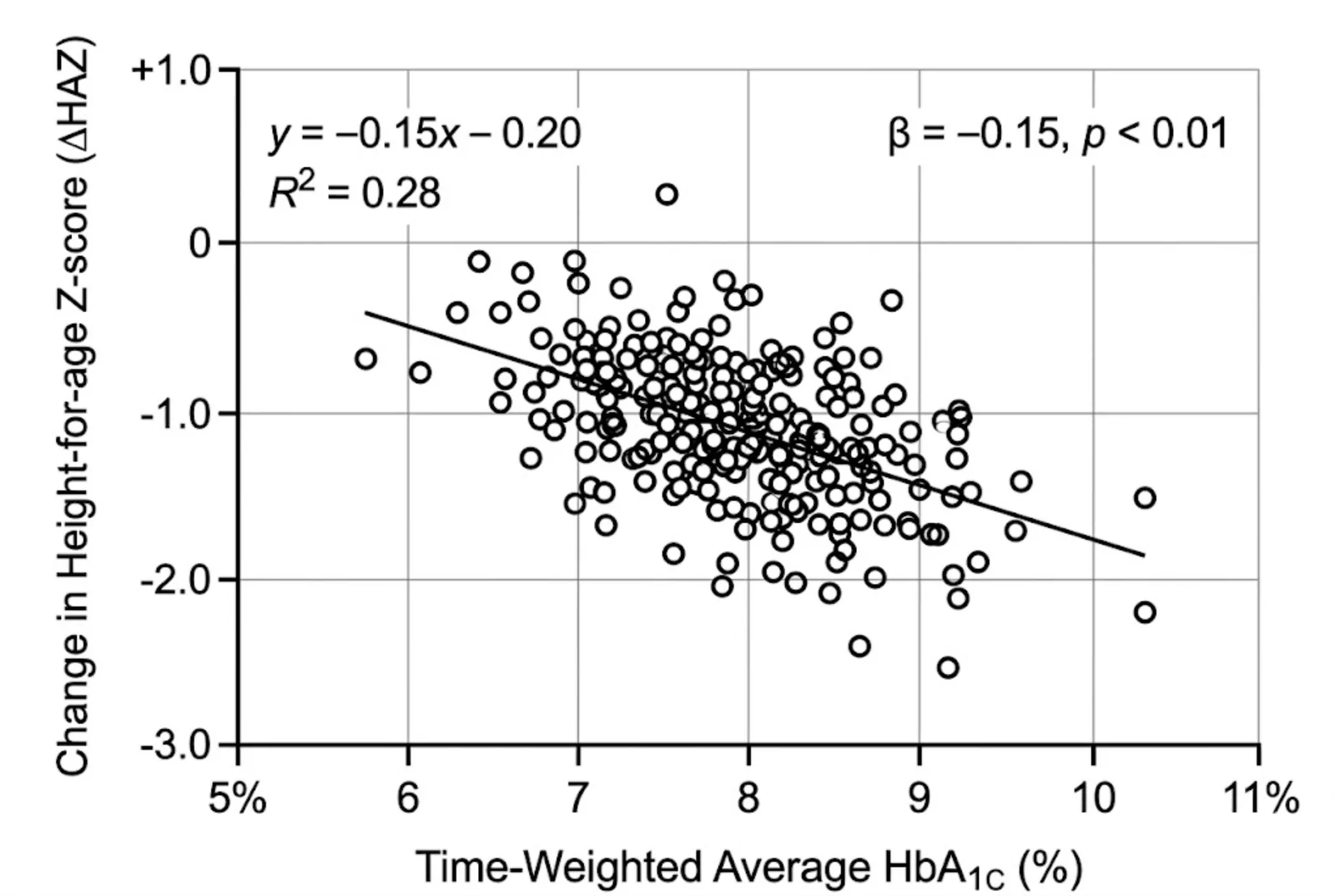
Scatterplot of time-weighted average HbA1c versus change in height-for-age z-score (ΔHAZ). This figure displays a scatterplot with each point representing one patient. The blue regression line shows a significant negative relationship (β = -0.15, p<0.01), indicating that higher time-weighted average HbA1c is associated with reduced linear growth. The shaded area represents the 95% confidence interval. HbA1c, glycated haemoglobin; HAZ, height-for-age z-score.

Positive associations were identified with ΔWAZ (β = 0.08, 95% CI: 0.01-0.15, p < 0.05) and ΔBMI-z (β = 0.10, 95% CI: 0.03-0.17, p < 0.01), suggesting that poorer glycaemic control was linked to disproportionate weight gain (Figure 3).

**Figure 3:**
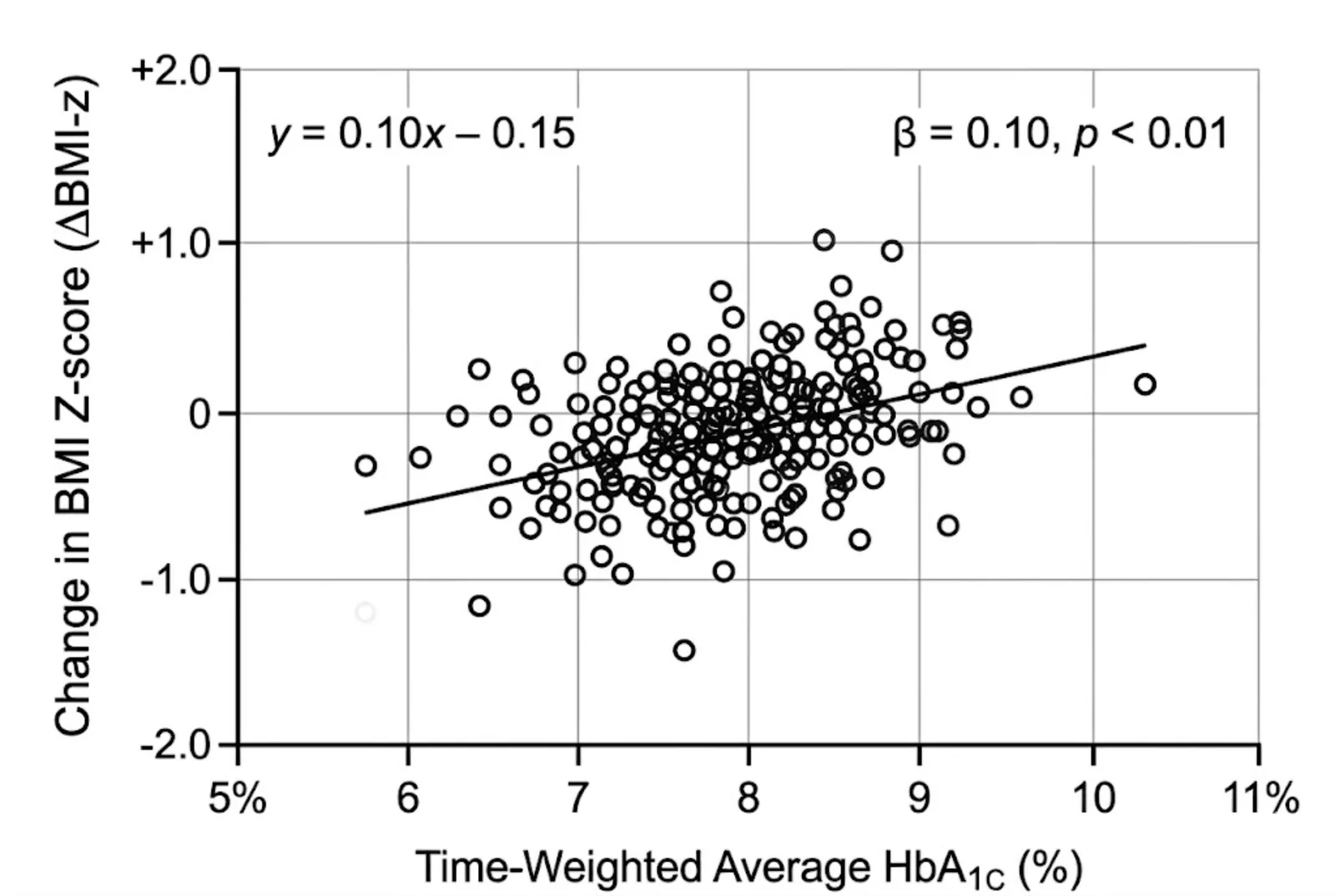
Scatterplot of time-weighted average HbA1c versus change in BMI z-score (ΔBMI-z). This figure displays a scatterplot showing a positive relationship (β = 0.10, p<0.01), indicating that higher cumulative HbA1c is associated with a greater increase in BMI. The shaded area represents the 95% confidence interval. HbA1c, glycated haemoglobin; BMI-z, body mass index z-score.

Figure 4 presents a forest plot summarising the beta coefficients for all auxological outcomes.

**Figure 4:**
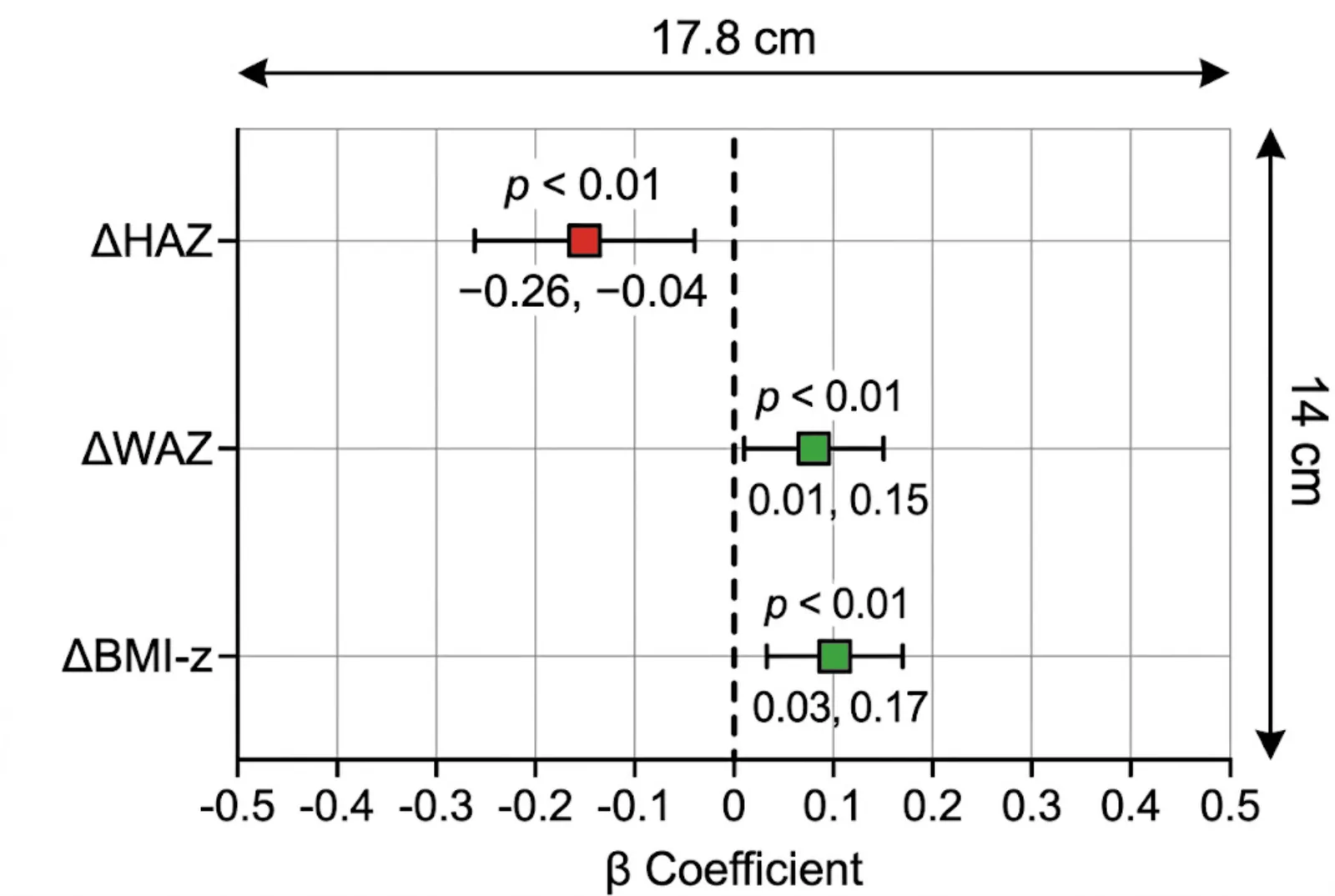
Forest plot of beta coefficients for auxological outcomes. This figure provides a visual summary of β coefficients and 95% confidence intervals for ΔHAZ (negative), ΔWAZ (positive), and ΔBMI-z (positive). The vertical line at β=0 represents no effect. CI, confidence interval; HAZ, height-for-age z-score; WAZ, weight-for-age z-score; BMI-z, body mass index z-score.

Subgroup findings

The negative effect on HAZ was most pronounced in children with diabetes duration greater than 5 years (β = -0.22, p=0.003) and in those with baseline HbA1c greater than 9% (β = -0.28, p<0.001), indicating that longer disease duration and poorer baseline control were associated with greater impairment in linear growth.

## Discussion

This longitudinal study of 200 children with type 1 diabetes demonstrates that early and sustained glycaemic control, measured by time-weighted average HbA1c, is associated with better linear growth and a more favourable weight profile. The inverse association with ΔHAZ (β = -0.15 per 1% HbA1c increase) is clinically meaningful; a child with an average HbA1c of 9.5% would lose approximately 0.45 SD in height compared to a child with 6.5% [13].

The positive associations with ΔWAZ and ΔBMI-z indicate that poorer control is linked to disproportionate weight gain. The potential mechanisms proposed to explain this association, including insulin-related weight gain, compensatory or defensive eating behaviours driven by hypoglycaemia avoidance, and the central role of insulin in modulating the growth hormone/IGF-1 axis, should be considered as hypotheses rather than conclusions. Because insulin dose, hypoglycaemia frequency, dietary intake, and physical activity were not systematically collected in this cohort, these mechanisms remain speculative and require evaluation in future studies with detailed metabolic and behavioural assessments [14, 15].

Comparison with existing studies

Our findings are consistent with and extend the existing literature on growth outcomes in children with type 1 diabetes. Liu et al. (2010) reported that children with type 1 diabetes and poor glycaemic control exhibited both impaired linear growth and accelerated weight gain, a pattern reflected in the higher BMI z-scores observed among youth with diabetes in the SEARCH for Diabetes in Youth study [6].

The landmark Diabetes Control and Complications Trial (DCCT) reported that intensive insulin therapy was associated with a significantly increased risk of severe weight gain as an adverse event of treatment [15, 16], and subsequent long-term follow-up confirmed that while intensive therapy reduced microvascular complications, it was accompanied by sustained weight gain, highlighting the complex relationship between glycaemic control and anthropometric outcomes [17].

Weber et al. further underscored the clinical relevance of body composition metrics beyond simple BMI, noting that fat and lean mass indices provide additional insight into metabolic risk in children and adolescents - a consideration highly pertinent to the divergent trajectories of declining linear growth and increasing adiposity observed in our cohort [18].

The ISPAD Clinical Practice Consensus Guidelines have emphasised that diabetes education and multidisciplinary care are fundamental to achieving optimal glycaemic control and preventing complications, which is particularly relevant for growth outcomes in children with early-onset type 1 diabetes [19, 20].

The Hvidøre Study Group on Childhood Diabetes reported that glycaemic control varied significantly across European centres, and that growth parameters were influenced by both metabolic control and the insulin regimen [21, 22].

This paradoxical pattern of declining linear growth combined with excess adiposity may reflect the combined effects of chronic hyperglycaemia on the growth hormone-IGF-1 axis and defensive eating behaviours driven by hypoglycaemia avoidance.

Our study extends these findings by utilising time-weighted average HbA1c as a measure of cumulative glycaemic exposure, providing a more comprehensive assessment of glycaemic burden than single-point HbA1c measurements. This approach, as recommended by Gerstein et al., captures the longitudinal impact of glycaemic control on growth outcomes more accurately [7].

Clinical implications

These findings have several important clinical implications. Firstly, routine monitoring of auxological parameters should be integrated into standard diabetes care for children, with growth trajectories serving as an indicator of overall metabolic control. Secondly, children with longer diabetes duration and higher baseline HbA1c represent a high-risk group that requires intensified monitoring and earlier intervention to optimise their growth outcomes. Thirdly, given the divergent effects of glycaemic control on height and weight, clinicians should be vigilant for the pattern of declining linear growth with concurrent weight gain and consider multidisciplinary interventions, including nutritional counselling and physical activity promotion, to address both growth and metabolic goals simultaneously.

Limitations

This study has several limitations. The retrospective, single-centre design may limit the generalisability of these findings. Pubertal staging was not available for all patients, and the use of inferred pubertal status may have introduced some misclassification. Insulin dose, hypoglycaemia frequency, dietary intake, and physical activity were not systematically collected, limiting our ability to evaluate the mechanisms underlying the observed associations. Residual confounding from unmeasured variables (e.g., dietary intake, physical activity, and socioeconomic status) may influence the observed associations. The relatively short follow-up period of 18 months precludes assessment of long-term outcomes. Missing data were handled using multiple imputation techniques. The observed changes in z-scores represent relative anthropometric trends rather than clinical diagnoses of stunting or obesity.

Future research directions

Future prospective, multicentre studies with longer follow-up are needed to confirm these findings. Studies incorporating detailed dietary and physical activity data, as well as objective measures of pubertal status, would provide a more comprehensive understanding of the relationship between glycaemic control and growth in children with type 1 diabetes. Long-term follow-up studies should assess whether early growth patterns predict adult height and metabolic outcomes, while randomised controlled trials should evaluate the effectiveness of targeted interventions for high-risk children with impaired growth trajectories.

## Conclusions

This study demonstrates that early and sustained glycaemic control is associated with better linear growth and a more favourable weight profile in children with type 1 diabetes, with poorer control associated with declining linear growth and disproportionate weight gain. These findings demonstrate the value of time-weighted HbA1c as a predictor of auxological outcomes, guiding early intervention and personalised treatment strategies. The pattern of declining linear growth with concurrent weight gain observed in children with poor glycaemic control emphasises the necessity of multidisciplinary care addressing both metabolic and growth goals. Children with longer diabetes duration and higher baseline HbA1c require intensified monitoring to optimise their growth outcomes. Further prospective research is needed to validate these findings and develop targeted interventions for this high-risk population.
